# Pemphigus: trigger and predisposing factors

**DOI:** 10.3389/fmed.2023.1326359

**Published:** 2023-12-27

**Authors:** Francesco Moro, Jo Linda Maria Sinagra, Adele Salemme, Luca Fania, Feliciana Mariotti, Anna Pira, Biagio Didona, Giovanni Di Zenzo

**Affiliations:** ^1^Molecular and Cell Biology Laboratory, Istituto Dermopatico dell’Immacolata (IDI)-IRCCS, Rome, Italy; ^2^Dermatology Clinic, Istituto Dermopatico dell’Immacolata (IDI)-IRCCS, Rome, Italy; ^3^Rare Diseases Unit, Istituto Dermopatico dell’Immacolata (IDI)-IRCCS, Rome, Italy

**Keywords:** pemphigus vulgaris, pemphigus foliaceous, autoimmune bullous disease, trigger factors, predisposing factors, etiopathogenesis

## Abstract

Pemphigus is a life-threatening autoimmune blistering disease affecting skin and mucous membranes. Despite its etiopathogenesis remains largely unknown, several trigger and predisposing factors have been reported. Pemphigus is caused by autoantibodies that target desmoglein 1 and desmoglein 3, impacting desmosome function. However, circulating autoantibodies are often the consequence of a precipitating factor that occurs in predisposed individuals. This review aims to describe and discuss almost all trigger and predisposing factors reported as possible or probable cause of the disease. Among the reported trigger factors that may induce or exacerbate pemphigus, we have found of particular interest: drug intake (especially thiol- and phenol-containing compounds), vaccines, infections, as well as some reports about pregnancy, radiations, emotional stress, pesticides and physical trauma. Moreover, we discuss the possible role of food intake in pemphigus onset and particular attention is given to dietary factors containing thiol, phenol and tannin compounds. A trigger factor is “the straw that breaks the camel’s back,” and often acts together with predisposing factors. Here we discuss how pemphigus onset may be influenced by genetic susceptibility and comorbidities like thyroid diseases, malignancies and other autoimmune disorders.

To identify other hitherto unknown trigger and predisposing factors, well designed prospective studies are needed. In this context, future research should explore their connection with the aim to advance our understanding of pemphigus pathogenesis.

## Introduction

Pemphigus disease, including pemphigus vulgaris (PV) and pemphigus foliaceous (PF), belongs to the intraepithelial autoimmune bullous disease (AIBD) group, affecting the skin and mucous membranes. Pemphigus is provoked by an altered desmosome function due to deposition of autoantibodies (autoAbs) directed against desmosomal components: desmoglein (Dsg) 3 and 1, leading to the acantholysis. PV is a potentially life-threatening disease characterized by flaccid cutaneous or mucosal bullae, that easily break and form painful erosions. In PV pathogenesis, autoAbs are directed against Dsg3 or both Dsg3 and Dsg1. PF is a less severe form of pemphigus. In PF the autoAbs are directed to Dsg1 which is localized throughout the epidermis. Dsg3 is expressed in basal and suprabasal layers of keratinocytes and compensates for detachment induced by anti-Dsg1 autoAbs that leads to loss of adhesion in the upper layers. As a consequence, PF is clinically characterized by crusty sores that often begin on the scalp and may also interest the chest, back, and face, while mucous membranes are frequently not involved. On the contrary in PV with only anti-Dsg3 antibodies blister formation occurs deep in the mucous membranes, where Dsg1 does not compensate for loss of Dsg3-mediated adhesion. While in case of developing of both anti-Dsg1 and anti-Dsg3 antibodies, the function of both Dsgs is affected and blisters develop in the skin and mucous membranes (1). Diagnosis is based on clinical assessment, histopathological examination and intercellular deposits of IgG and/or C3 by direct immunofluorescence. Circulating autoAbs assessed by indirect immunofluorescence and/or Dsg3 and/or Dsg1 ELISA have a confirmatory value.

The role of anti-Dsg autoantibodies in PV pathogenesis has been largely demonstrated. Titers and profiles of anti-Dsg antibodies have been correlated with disease activity and clinical phenotype. In addition, passive transfer of IgG from pemphigus patients’ results in pemphigus-like lesions in mice. Moreover, adoptive transfer of splenocytes from Dsg3-knockout mice immunized with murine Dsg3 induced PV phenotype into immunodeficient mice (2). An autoreactive B cell response mainly directed to Dsg 1 and Dsg3 is sustained by a T cell response that is also crucial for pemphigus onset (3). However, the mechanism behind the loss of B and T-cell tolerance is not completely understood so far (4).

Pemphigus is a prototype of an organ-specific autoimmune disease and most agents that favor immune system stimulation may be susceptible to provoke the disease in genetically predisposed individuals.

The etiopathogenesis of pemphigus is largely unknown. Several trigger factors have been described to induce or exacerbate pemphigus, such as drugs, vaccines, pregnancy, radiations, emotional stress, infections, diet or other external factors. A trigger factor is “the straw that breaks the camel’s back,” and acts together with predisposing factors, such as genetic susceptibility and comorbidities.

The aim of the present review is to highlight the trigger and predisposing factors possibly involved in the etiopathogenesis of this AIBD.

Some trigger factors reported in this review are not based on enough evidence, but we choose to report them to possibly inspire other studies that could confirm or disprove their possible role in the onset of pemphigus.

To identify other hitherto unknown trigger and predisposing factors well designed prospective studies should be conducted.

## Trigger factors

### Drugs

**Figure 1:**
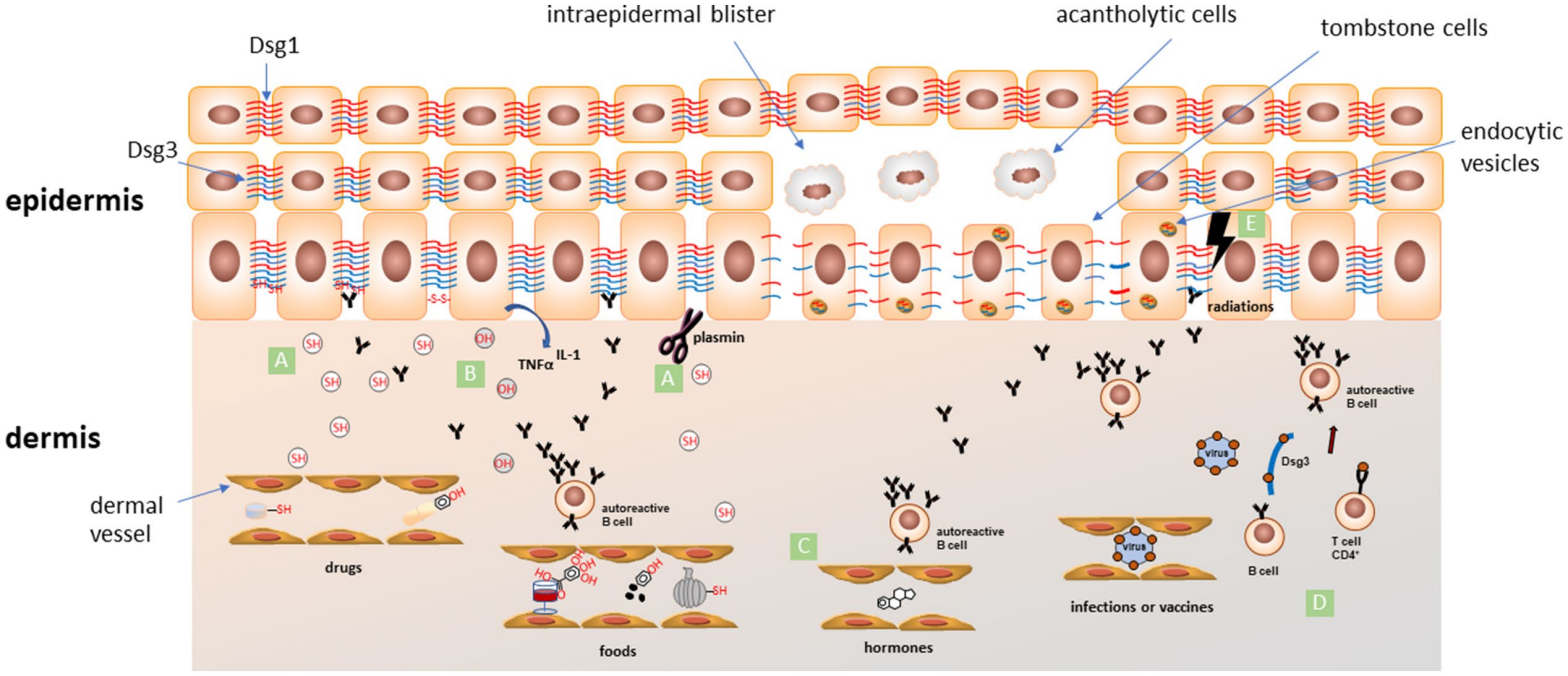
Schematic representation of proposed mechanisms for the most accepted pemphigus trigger factors. **(A)** The thiol-containing drugs and foods (SH) can induce acantholysis by different pathways: (i) interacting with Dsg 1 and 3, they could form neo-epitopes, promoting an autoimmune response; (ii) sulfhydryl groups involve keratinocytes in a disulfide bond altering membrane adhesion; (iii) activation of proteolytic enzymes such as plasmin that could directly cleave adhesion proteins or indirectly inhibit adhesion process; **(B)** phenolic compounds (OH) in drugs and foods can induce the release of pro-inflammatory cytokines like TNF-α and IL-1 from keratinocytes. These cytokines are involved in recruitment of immune cells and activation of plasminogen and other proteases involved in the acantholytic process; **(C)** estrogens levels during pregnancy or menstrual cycle can favor an alteration in Th1/Th2 balance toward a Th2 response, followed by an increase of IgG autoAbs production; **(D)** an immune response targeting microbial antigens coming from infections or generated after vaccination can cross-react (molecular mimicry) with some epitopes of endogenous molecules (such as Dsg3) leading to activation of CD4+ T cells and initiation of the autoimmune cascade; **(E)** ionizing radiations could alter skin antigen expression, unmasking hidden epitopes and inducing an autoimmune response in predisposed individuals. All these factors can interfere with normal Dsgs network, induce endocytosis of Dsgs (endocytic vesicles) and activate different intracellular signalling pathways leading to loss of keratinocyte adhesion and acantholysis. Dsg1 in red, Dsg3 in blue.

Drugs are considered the most common trigger factors for pemphigus disease (5). They could be divided into three groups according to their dominant chemical structure: thiol drugs, phenol drugs, and non-thiol/non-phenol drugs (5). In a systematic review conducted on 170 patients, the most reported drugs related to pemphigus onset are penicillamine (33.1%), captopril (7.7%), and bucillamine (6.5%). Other involved drugs are: ingenol mebutate, cilazapril, metamizole (dipyrone), imiquimod, penicillin, fosinopril, diazinon, glibenclamide, carbamazepine, lisinopril, nifedipine, rifampin, gold sodium thiomalate, ceftazidime, chloroquine/hydroxychloroquine (4–28) (Table 1). Thiol drugs contain a sulfhydryl group (-SH), and are the most common medications inducing pemphigus. These drugs induce acantholysis by different pathways. On one side the sulfhydryl group involves keratinocytes in a disulfide bond, altering membrane adhesion. On the other one, they activate proteolytic enzymes such as plasmin and inhibit enzymes that promote keratinocytes adhesion (29). Moreover, interacting with Dsg 1 and 3, they could form a neo-antigen, promoting autoimmune response. The most important thiols reported are penicillamine, captopril, and tiopronine (29) (Figure 1).

**Table 1:** List of trigger factors.

Drugs	Vaccines	Infections	Nutrition	Other factors
ACE inhibitors Cilazapril Fosinopril Lisinopril Captopril Enalapril Benazepril Quinapril Ramipril NSAID Acetylsalicylic acid Metamizole Angiotensin receptor blocker Losartan Irbesartan Clacium-channels blockers Nifedipine Biologic drugs Secukinumab, Tocilizumab Other drugs Imiquimod Carbamazepine Glibenclamide Ceftazidime Hydrochlorothiazide Hydroxycloroquine Ingenol Mebutate Rifampin Levodopa Heroin Penicillamine Gold Sodium Thiomalate Penicillin Bucillamine Oral contraception 5-aminolaevulinic acid-based photodynamic therapy	Influenza Hepatitis B Rabies Tetanus COVID 19 vaccines Comirnaty Vaxzevria Spikevax ChAdOx1 nCoV-19 Sinopharm COVID-19	Human herpes virus HHV HHV8 Herpes simplex virus Citomegalovirus Epstein Barr virus Hepatitis B virus Hepatitis C virus Human immunodeficiency virus Rotavirus *Helicobacter pylori*	Thiols Garlic, leek, chives, onion, shallot Phenols Mango, cashew nuts, black peppers, red chillies Tannins Mango, cassava, yucca, guarana, betel nut, raspberry, cranberry, blackberry, avocado, peach, ginger, tea, ginseng, red wine Isothiocyanates Mustard oil Phycocyanin Walnut proteins	Pregnancy Radiations Emotional stress Pesticides Organophosphates Organochlorines Traumas Surgery Accidental traumas Electrical injury Bee sting Chemicals Photographic developing Dry cleaning Industrial solvent Phenol

The chemical structure of phenol drugs is based on an aromatic hydrocarbon group bonded with a hydroxyl group (-OH). These drugs induce the release of pro-inflammatory cytokines like tumor necrosis factor alpha (TNF-α) and interleukin (IL)-1 from keratinocytes. These cytokines are involved in the activation of plasminogen and other proteases and complement, all involved in the acantholytic process (Figure 1). The most important phenol compounds triggering pemphigus are aspirin, heroin, rifampin, and levodopa (29, 30).

Non-thiol/non-phenol drugs could also induce pemphigus through different signalling pathways that include antigen modification, autoAbs induction or immunomodulation. The most noteworthy non thiol/non phenol drugs are non-steroidal anti-inflammatory drugs, and calcium-channel blockers (29).

Drugs can also induce pemphigus through the production of immunoglobulin (Ig) G autoAb against Dsg-1 and 3, provoking an immunologic acantholysis (31).

It has been reported that thiol-containing drugs are more often associated with PF while non-thiol drugs with PV (32). Yoshimura and coworkers in a clinical and histopathological study on17 patients with drug-induced pemphigus, found that most of them showed a PF-type phenotype with antiDsg-1 autoAbs, caused by thiol-containing drugs (33). A paradoxical reaction with disease exacerbation after treatment with rituximab has been reported, but more studies are needed to better elucidate this finding (34, 35).

Hayashida and coworkers reported a drug-induced pemphigus in a patient affected by rheumatoid arthritis treated with secukinumab, an anti-IL17 monoclonal antibody (mAb), then exacerbated after treatment with tocilizumab, an IL-6 receptor antagonist, both belonging to the non-thiol and non-phenol-group (36).

In 2018 Palleria and coworkers reported 3 cases of PV following treatment with ramipril, losartan, irbesartan and hydrochlorothiazide, drugs belonging to the families of angiotensin converting enzyme inhibitors (ACEi) and angiotensin II receptor blockers. ACEis represent the drugs most frequently associated with PV development (Table 1). Since 1980, a large number of PVs following therapy with ACEi and angiotensin receptor blockers have been reported. In particular, captopril seems to be the most involved, probably because of the sulfhydril group within its molecular structure. Other drugs involved are enalapril, lisinopril, benazepril hydrochloride, fosinopril sodium, quinapril hydrochloride, and ramipril (37) (Table 1).

In 2017, Zhou and colleagues documented a case of PV following treatment with 5-aminolaevulinic acid-based photodynamic therapy (ALA-PDT). While the role of UV radiation in the PV onset has been recognized for some time, this represents, to the best of our knowledge, the first report of PV development after ALA-PDT therapy (38) (Table 1). A case of imiquimod-induced PV has recently been reported. Imiquimod, as toll-like receptor-7 agonist, might induce pemphigus by stimulating dendritic cells and keratinocytes in overproduction of interferon (IFN)-alpha with consequent induction and maintenance of autoreactive B-cells (39).

Furthermore, in women with pemphigus a significantly higher use of oral contraception has been reported compared to the control group (40). This could be explained by the activation of some pathogenic signaling pathways by oral contraceptives. On the other hand, women with pemphigus could use more oral contraceptives compared to healthy individuals to prevent risky pregnancies (5).

Alternative medicine could be also a trigger for pemphigus. Yoo and coworkers reported a case of PF developed after a bee-venom acupuncture treatment and supposed that immunological stimulation by bee venom could have induced PF (41) (Table 1).

### Vaccines

Different vaccines against influenza, hepatitis B, rabies, and tetanus have been reported to induce or exacerbate pemphigus. However, considering the higher risk of infection in these patients and the immunosuppressive therapy necessary to treat the disease, vaccination against seasonal influenza, H1N1, tetanus, and pneumococci is still recommended (42–47).

In this context, vaccines against coronavirus disease (COVID)-19 have also been reported to trigger pemphigus. A recent review by Pira and coworkers shows that in the past 3 years, since the start of COVID-19 vaccinations, more than 15 cases of vaccine-induced PV and more than 7 cases of vaccine-induced PF have been reported. Patients of both genders, ages 30 to 89, developed pemphigus 5 to 30 days after the first or second dose of vaccine. The involved vaccines are Comirnaty (BNT162b2), Vaxzevria, Spikevax, ChAdOx1 nCoV-19 vaccine and Sinopharm COVID-19 (BBIBP-CorV). It could be hypothesized that the vaccine acts as a precipitating factor inducing autoimmunity in genetically susceptible individuals by stimulating pre-existing and subclinical autoreactivity against PV targets (48) (Table 1).

### Pregnancy

Pregnancy could be a trigger factor for pemphigus (49) (Table 1)_._ Many cases of flare-up during pregnancy followed by a remission after delivery have been described (50). This could be explained by the rapid increase in estrogens levels during pregnancy, that favour an alteration in Th1/Th2 balance toward a Th2 response, followed by an increase of IgG autoAbs production (Figure 1). It is very important to manage this autoimmune disease during pregnancy and to prevent its onset in genetically predisposed patients (51). Indeed, control of disease activity, the choice of an appropriate treatment with reasonable few side effects, the strict follow-up of serological parameters and of clinical manifestations, and the study of genetic predisposition are crucial points to guarantee a safe pregnancy in pemphigus female patients.

### Radiations

Development of pemphigus lesions after radiotherapy has been described (Table 1). In a recent case–control study on 365 patients with AIBDs, Hung and coworkers found that 53.4% of the cases had an exposure to radiotherapy before skin disease onset vs. 33.1% of controls, and that this relation was particularly strong in patients with breast cancer (OR 2,986) (52). Unfortunately, authors do not differentiate PV from bullous pemphigoid patients, and do not report specific data about the association between radiotherapy and PV.

Until now, only 30 anecdotal cases were described (53). In reported cases, which have ages ranging from 37 to 92 years (median age: 62 years), lesions seem to appear within variable times (1 week to a year, mean time 3 months) after radiotherapy (52, 54). In 90% of patients lesions appear on irradiated area, and progress to non-irradiated skin in 80% (53). No relationship with radiation dose was observed, as the minimum reported inducing dose was 38G (54). As a pathogenetic hypothesis, ionizing radiation could alter skin antigen expression, unmasking certain epitopes, thus inducing autoimmune response in predisposed individuals (52) (Figure 1). Interestingly, Robbins et al. described a patient with only circulating anti-Dsg3, that developed PV lesions on the irradiated area (53, 55). Intralesional immunomapping of Dsg1, showed an altered expression, suggesting a role for ionizing radiation in altering Dsg1 expression and triggering lesions onset (Figure 1).

Radiotherapy-associated pemphigus seems to have a severe course and usually needs high-dose corticosteroids to get into remission. Rituximab has been successfully adopted in some resistant cases (54).

Ultraviolet (UV) radiation could be another trigger for pemphigus onset. In a recent study on a series of non-endemic PF patients in Turkey, the authors reported a higher incidence of pemphigus onset and relapses during the spring–summer period. This seasonal feature leads the authors to assume that UV could have a role in the pathobiology of the PF disease by inducing the acantholysis (56). An article by Aghassi et al. reported a case of PF following therapy with Psoralen–UV-A (PUVA). Subsequently, a case report was published regarding a case of herpetiform pemphigus that developed following UV-B therapy in a patient with psoriasis. In these patients, there could be a synergistic effect in pemphigus onset, dictated by the underlying condition for which the therapy is administered, such as psoriasis, and the therapeutic intervention with PUVA and UV-B (57, 58).

### Other external factors

Traumas have been reported as a triggering factor for pemphigus in a limited number of patients (59) (Table 1). The largest series, described by Daneshpazhooh and coworkers includes 36 patients that developed pemphigus lesions after traumatic events like surgical (dental, orthopaedic or abdominal) procedures or accidental traumas. Thirteen patients had a new-onset pemphigus. Lesions developed after a short time (from 1 week to 1.5 month) from trauma exposure (59). Electrical injury has also been reported as a possible triggering factor. In a case report PV developed with recurrent oral ulcerations 1 year after the electrical injury (60). This could be explained as a gradual alteration in the self-antigen recognitions. Bee stings could be another possible triggering factor due to the cytokine concentration in the sting site (61).

Pesticides could have a critical role in pemphigus onset (40, 62–66). Pietkiewicz and coworkers in 2017 reported 3 cases of pemphigus in a cluster population living near a wastewater treatment plant. They hypothesized a possible role of topical absorption of chemical compounds and emotional stress on the pemphigus onset (67) (Table 1). Alteration of estrogen dependent pathways induced by organochlorine pesticides could promote the production of Th2 cytokines, leading to B-cell mediated autoimmunity (68)^,^ (69). Another suggested pathway is the loss of cell adhesion as a consequence of decreased skin muscarinic and nicotinic receptors expression (70). In a recent study, authors measured pesticides in hair samples of pemphigus patients and healthy controls confirming different distribution of contamination with organophosphates and/or organochlorines in hair samples in PV and PF and controls (6.3, 25.6, and 11.9%, respectively; *p* = 0.0437) (71). More conclusive data come from a very recent systematic review and meta-analysis from Chang and coworkers. They have demonstrated that exposure to pesticides was significantly associated with pemphigus development. Suggested mechanism includes direct damage to cell adhesion molecules by pesticides, and the balance of Th1 and Th2 cells skewed towards the Th2 profile, with generation of autoAbs (72).

There are several evidences supporting the role of contact dermatitis caused by chemicals, photographic developing liquids, dry cleaning, industrial solvents and other molecules in pemphigus onset (73).

Specifically, it has been shown that contact with phenols can induce pemphigus topically. Pemphigus developed in a 66-years-old woman after a cosmetic skin procedure in which phenol-containing chemical peels were used (74). In a more recent case report, pemphigus developed in a 32-years-old woman as a result of exposure to a nonyl phenol containing cleaning agent (75). The authors reported also several other studies with cases of pemphigus induced by contact with various substances such as garlic, benzoin tincture, basochrome, diclofenac.

Finally, an investigative study has shown increased levels of Lachnospiracea incertae sedis, Coprococcus, and Flavonifractor in the gut of pemphigus patients suggesting a role of specific microorganisms in the induction of the disease (76).

### Emotional stress

Emotional stress in patients with family history of pemphigus or genetic susceptibility could lead to the onset or the exacerbation of blistering (77–79) (Table 1). Emotional stress could induce the initiation of different signalling pathways such as glucocorticoid, leading to an alteration of cytokine production. In 1961 Perry and Brunsting described for the first time the association of emotional stress and pemphigus (80). Actually, several reports discussed the role of emotional stress in the induction or exacerbation of pemphigus disease, mainly in genetically predisposed patients (77–79). This could be due to an alteration of the signalling pathways provoked by stress, such as the glucocorticoid hormone secretion that lead to an alteration of the cytokine secretion. Recently, Wei and colleagues reported 24 cases of pemphigus, of which about 1 in 6 were found to have post-traumatic stress disorder, a prevalence comparable to that of post-traumatic stress disorder following a cancer diagnosis and three times higher than that of the general population. This study highlights how the relationship between pemphigus and emotional stress may exist in both directions. (81). Therefore, a psychiatric assessment in pemphigus patients could be recommended to prevent a worsening of the autoimmune disease (82). However, besides pemphigus, emotional stress could have a role in other autoimmune diseases (83).

### Infections

Association between pemphigus and viral infections is discussed by many authors (62, 84–90). Viral infections can complicate the treatment of pemphigus, postponing the immunosuppressive therapy to avoid a reactivation of the infectious disease (Table 1). There are many ways a viral infection can induce cutaneous autoimmunity. The first and more reasonable is the molecular mimicry between the viral proteins and the molecules expressed by epidermal cells. After the infection, antigen presenting cells process the viral fragments inducing an immunologic overactivation against self-antigens (Figure 1). Inflammation resulting from infection as well as the viral infection itself can induce cell modification and tissue damage, leading to the unveiling of previous unknown epitopes that can stimulate an autoimmune response. Virus can directly infect B cell and induce polyclonal activation, proliferation and increasing antibodies production. Virus can also influence T lymphocytes inducing their polyclonal activation with superantigens. Superantigens bind both major histocompatibility complex (MHC) class II and T cell receptors and affect signalling pathways resulting in cytokines production and polyclonal T cell proliferation that could result in an autoimmune response. Moreover, silent autoreactive T-cells can be stimulated by inflammation and cytokines during a viral infection and their proliferation can lead to an autoimmune response (89). While contemplating viruses and viral diseases as a possible trigger factor for pemphigus Ruocco and coworkers also consider their pharmacological treatment (91). In fact, immunomodulatory therapies such as IFN and other cytokines have been associated with AIBDs onset and reactivation or exacerbation of an autoimmune disease like pemphigus in genetic susceptible subjects (91). Human herpes virus (HHV) is the virus family more often associated with pemphigus onset. Considerable evidence linking herpes simplex virus (HSV) infection and pemphigus has been highlighted by many authors studying patient serology for HSV and finding HSV DNA in skin lesions (83, 84, 88–103). HHV-8 has been linked to pemphigus disease also in patients without human immunodeficiency virus (HIV) or Kaposi sarcoma, finding specific HHV-8 DNA in patients skin biopsy and specific IgG in patients serum (104, 105). However other authors did not find this linkage between HHV-8 and pemphigus not being able to demonstrate the presence of HHV-8 DNA in patients skin lesions (106–108). The development of PF after citomegalovirus infection has been described in a case report of a child with genetic susceptibility for the disease (109). A possible explanation of interaction between infection and PV is the molecular mimicry. In this context, Cho et al., identified two cross-reactive VH1-46 Abs that both disrupt keratinocyte adhesion and inhibit rotavirus VP6 replication suggesting that in the B cell population some clone, through somatic mutation, may become specific to both Dsg3 and VP6 (110). They also showed that Dsg3-specific memory B cells collected in a PV patient prior to disease diagnosis presented an activated phenotype and signs of ongoing affinity maturation. This gradual process could be at the base of induction of clinical visible disease and could start also from a immune response to viral infection (111). The association between Epstein–Barr virus (EBV) and pemphigus has been investigated by some authors (84, 112, 113). EBV DNA has been found in pemphigus patients skin biopsy and elevated EBV IgG titers in peripheral blood, suggesting a link between EBV infections and the onset of pemphigus (84). Several authors investigated the possible link between hepatitis B and C virus and pemphigus (90, 114–116). In a population based study, Kridin and coworkers found that pemphigus patients have a higher prevalence of hepatitis B virus chronic infection than the controls, although no significant difference was detected for hepatitis C virus chronic infection (90). However, a retrospective study including 62 pemphigus patients and 50 controls detected no significant association between hepatitis virus infections and pemphigus (114).

PV associated with HIV infection has been described in 6 case reports (117). In four of six HIV preceded PV (118–121), in one HIV and PV were diagnosed concurrently (122) and in another one PV preceded HIV diagnosis (123). Ruocco and coworkers reported a case of PV onset 2 weeks after a coxsackievirus infection treated with cefixime (cephalosporin) (124). Pemphigus onset was described as a paraviral eruption as a consequence of both virus and drug effects on the immune response (91, 124).

Finally as for bacterial infections Mortazavi and coworkers showed that untreated PV patients had significantly higher IgG positivity to *Helicobacter pylori* compared with the healthy individuals (79.3% vs. 59.5%, *p* = 0.004) suggesting that these pathogenic agents may contribute to PV pathogenesis (125). Cutaneous manifestation of staphylococcal scalded skin syndrome could mimic PF due to a toxin, produced by the *Staphylococcus aureus*, which targets Dsg1 in the skin layer (126).

### Nutrition

A growing body of evidence shows that some nutrients are involved in modulating immune responses and contribute to the pathogenesis of several cutaneous disorders, including bullous diseases (127, 128) (Table 1). A variety of dietary factors has been proposed to play important roles in the onset, progression, exacerbation and treatment of these diseases.

Dietary factors have been suggested to be involved in pemphigus induction based on the similarity of their chemical structure to drugs recognized as possible trigger factors (129, 130). Clinical evidence supports the role of dietary factors in the maintenance and exacerbation of pemphigus (131, 132). Suspected dietary factors contain thiol compounds (garlic, leek, chives, onion, shallot), phenols (black pepper, red chillies, mango, cashew), tannins (tea, red wine, spices, raspberry, cranberry, blackberry), isothiocyanates (mustard, horseradish, cauliflower) and phycocyanins (*Spirulina platensis* alga) (127–132).

### Thiols

In a case report, heavy garlic consumption worsened pemphigus in a 49-year-old man (131): a garlic-free diet coincided with remission for a few months, followed by recurrence after unintentional ingestion of a garlic-spiced meal. In another case report, oral lesions of pemphigus were induced by ingestion of leek (132) (Table 1). A leek-free diet resulted in oral lesions improvement and antibody titers decrease, while a leek challenge induced oral lesions along with increased antibody titers (132). An *in vitro* study showed that three compounds of garlic (allylmercaptan, allylmethylsulfide and allylsulfide) induced acantholysis in skin specimens in four of seven donors cultured in the presence of each of the allyl compounds for 3 days (133).

Since thiol compounds in food have been suggested to be involved in the induction of pemphigus based on the similarity of their chemical structure to drugs, a number of mechanisms have been suggested for the effect of thiols: direct biochemical effect by formation of thiol-cysteine bonds instead of cysteine-cysteine bonds, and disturbance to cell adhesion; inhibition of enzymes that aggregate keratinocytes; activation of enzymes that disaggregate keratinocytes like plasminogen activator; immunological reaction with a formation of a neoantigen (134). The antibodies against the new complex have a cross reaction with desmosomes and provoke pemphigus disease (135).

### Phenols

The early age of onset and high incidence of pemphigus in Indian patients might be explained by the high consumption of foods that contain large amounts of phenols, such as mango, cashew nuts and black peppers (128, 136) (Table 1). However, it must be considered, when examining specific populations, the potential impact of genetic factors on the development of the pathology, factors that could downscale the relevance of external risk factors, such as those related to specific dietary habits (137).

Possible mechanisms of phenol-induced pemphigus include the induction of IL-1α and TNF-α release by keratinocytes (138). These cytokines have been shown to be relevant in the regulation and synthesis of complement and proteases, like plasminogen activator, which has been implicated in the pathogenesis of acantholysis in PV (139).

### Tannins

Several foods and drinks contain tannins such as mango, cassava, yucca, guarana, betel nut, raspberry, cranberry, blackberry, avocado, peach, ginger, ginseng, tea and red wine. Although there have been no reports of tannin-containing foods directly inducing pemphigus, some *in vitro* studies have linked tannins with skin acantholysis (140, 141). In one report, tannic acid added to *in vitro* cultured human breast skin explants from five different donors without any bullous disease, produced different cytotoxic effects, including suprabasal cleavage and intraepidermal acantholysis (140); the concentrations required to achieve these effects varied significantly between different samples, suggesting high variability in susceptibility to tannin acantholysis. Feliciani and coworkers used high-performance liquid chromatography (HPLC) to measure the levels of tannic acid in the skin blister fluid of four group of patients, subdivided according to their dietary habits (141). Patients with a tannin-rich diet had increased tannin metabolites in their skin. In the same study, in a keratinocyte cell culture experiment, tannic acid was capable of inducing acantholysis, effect that decreased when anti-IL-1α and anti-TNF-α antibodies were added (141). Foods with large amounts of tannins are also heavily consumed in India, another area with a high incidence of pemphigus (128).

### Isothiocyanates

No cases of pemphigus induction or aggravation through the consumption of foods containing isothiocyanates, derived from hydrolysis of glucosinolates have been reported so far. These foods may contain an allyl, benzyl or phenyl group and can be immunologically active or lead to non-immunologic skin acantholysis, like thiol-containing drugs. Allyl isothiocyanate is the primary constituent of mustard oil, a known irritant that can cause blistering of mucous membranes (142). Tur and Brenner noted that mustard oil is widely used in India, where pemphigus has a high incidence, not only as a food but also for topical application on scalp hair and for body massage. Contact with this oil may cause antigen exposure leading to pemphigus or other local effects (136). Mustard is a member of the Cruciferae family; other members include horseradish, winter cress, turnip, radish, cabbage, cauliflower and broccoli, but to date no clinical cases are reported.

### Phycocyanin

Phycocyanin is the blue pigment protein in blue-green algae such as *Spirulina platensis,* which is sold in food stores as a dietary supplement. Two reports have suggested a link between intake of herbal supplements and bullous disorders. In the first report, the authors have described two cases. The first case is a 57-year-old man with chronic PV, which was controlled with azathioprine and prednisone, who experienced a severe flare after intake of a mixture of dietary supplements containing *S. platensis*; the flare resolved in 2 weeks, upon discontinuation of these supplements, which had never been taken before this episode, and an increased in his prednisone dose (143). The other case was a 55-year-old man with PV treated and controlled with prednisone, dapsone and azathioprine, who developed blisters on his trunk, head and oral mucosa within 1 week of starting to take an *Echinacea* supplement daily. After discontinuing the use of the *Echinacea* supplement, partial disease control, but never complete remission, was achieved with prednisone, azathioprine and dapsone (143). In these two patients the temporal relationship of a PV flare occurring within days of ingesting these herbal supplements is highly suggestive of a causal relationship, but it cannot exclude the possibility that these patients may have experienced a flare in conjunction with a standard prednisone taper.

In the second report, a mixed immunobullous disorder with features of both PF and bullous pemphigoid developed in an 82-year-old healthy woman 1 year after she started a food supplement containing the blue-green alga *S. patensis* (144). The patient’s symptoms and signs resolved 3 months after completion of a prednisone treatment and avoidance of the dietary supplement.

### Gluten

In a report, two patients with new-onset pemphigus, one with IgA pemphigus and one with PF, started a gluten-free diet with subsequent complete remission of their symptoms and signs (145). Both patients had serologic markers of gluten-sensitive enteropathy (IgA and IgG versus antigliadin antibodies) without any manifestations of celiac disease, suggesting that they had silent gluten sensitivity. From these findings, it is plausible to hypothesize an association between gluten intake and pemphigus, and how individuals with pemphigus and serologic markers of gluten-sensitive enteropathy may benefit from a gluten-free diet.

### Walnut antigens

In a recent study, it has been hypothesized that walnut antigens can trigger autoAb development in patients with PV through a “hit-and-run” mechanism (146). Revertant/germline mAbs of 8 anti-Dsg3 pathogenic mAbs from PV patients were tested for reactivity against a panel of possible allergens, including food, epithelia, insects, pollens, and fungi antigens. Lin and coworkers showed that all tested germline PV mAbs reacted to walnut antigen extract, specifically to two protein components, Jug-R2 and uncharacterized 85-kDa protein, regardless of their reactivity to Dsg3 autoantigen. This suggests that walnut proteins might be exogenous antigens that activate naive B cells in subjects genetically predisposed to PV, leading to subsequent autoAb development and disease onset. So walnut antigen might be the initial stimulus and selection of autoreactive B cells is subsequently driven by reactivity to Dsg3. Indeed, IgG1 and IgG4 antibodies against walnut antigens are present at much lower levels than those against Dsg3 in sera from patients with PV (146).

## Predisposing factors

### Genetic susceptibility

Pemphigus, like many other autoimmune diseases, is strictly related to immune responses that could be altered via gene polymorphisms (Table 2). Differences in the incidence of pemphigus in diverse ethnic populations, some of which could present a higher incidence or endemic distribution (i.e., PV in Ashkenazi Jewish), strongly suggest a role of genetic factors. Furthermore, familial pemphigus cases have even been reported. MHC genes, called human leucocyte antigens (HLA) in humans, are the most involved genetic factors in pemphigus (147, 148). Several studies regarding genetic predisposition reported the association of pemphigus with the class II HLA alleles in specific ethnic groups (149–151). Among Italian, Spanish, French, Slovak, North American, and Brazilian PV populations, the most common associated alleles are DQA1*01:04, 03:01, DQB1*05:03 and DRB1*04:02, 14:01 (152–159). Yan and coworkers reported in a meta-analysis that HLA-DRB1*04, HLA-DRB1*14, and HLA-DRB1*08 HLA are statistically important susceptibility factors for PV, while in the Jewish population, an association between PV and HLA-DRB1*04:02, and DQB1*03:02 has been reported (148, 149). MHC region includes also some long non-coding RNA (lncRNA) genes in the HLA complex group (HCG): recently, Salviano-Silva and coworkers, found an association between HCG lncRNA alleles and pemphigus susceptibility, suggesting their role in pemphigus pathogenesis (160) (Table 2).

**Table 2:** list of predisposing factors.

Genetic susceptibility	Comorbidities: thyroid diseases	Comorbidities: malignancies	Comorbidities: autoimmune disorders
HLA-DRB1*04:02 HLA-DRB1*14:01 HLA-DRB1*08 HLA-DQA1*03:01 HLA-DQA1*01:04 HLA-DQB1*05:03 HLA-DQB1*03:02 SNPs of ST18 SNPs of FOXP3 SNPs of cytokines (IL-4, IL-6, IL-10, TNFα, TGFβ) lncRNA in HCG	Hypothyroidism Hashimoto’s thyroiditis	Hematologic malignancies Leukemia Multiple myeloma Non-Hodgkin lymphoma Internal malignancies Kaposi’s sarcoma Oropharyngeal cancer Gastrointestinal cancer Colon cancer Laryngeal cancer Esophageus cancer	Lichen planus Pemphigoid Alopecia areata Rheumatoid arthritis Systemic lupus erythematosus Psoriasis Autoimmune thyroiditis

HLA, human leukocyte antigen; SNP, single nucleotide polymorphism; lncRNA, long non-coding RNA genes; HCG, HLA complex group.

On the other hand, it has been demonstrated, through a genome-wide association study in the Jewish population, an association between single nucleotide polymorphisms (SNPs) of the ST18 gene and PV (161). ST18, a regulator of apoptosis and inflammation overexpressed in the skin of PV patients compared to healthy individuals, has been hypothesized to have a role in the pathogenesis of pemphigus provoking an elevated production of TNF-α, IL-1α, and IL-6 and favoring a PV IgG-induced cell–cell altered adhesion (162) Although in Egyptian, Jewish, and Iranian populations SNPs of ST18 and PV association was reported, in Italian (163), Chinese and German population no linkage between ST18 SNPs and PV was demonstrated (161, 164, 165). In addition, increased CD59 transcriptional levels have been associated with gene expression, mainly in female subjects, in PF (166). In this context, a study underlines the importance of a SNP in forkhead box P3 (FOXP3) gene for the development and function of regulatory T-cell, describing SNP association with the clinical course and prognosis of PF (167) (Table 2).

Furthermore, polymorphisms of genes encoding several cytokines, such as IL-4, IL-6, IL-10, TNF-α, and transforming growth factor-β have been reported in pemphigus patients compared to healthy controls (168–174), suggesting a potential involvement in the pathogenesis of this disease.

### Comorbidities

Comorbidities can be considered as a predisposing factor because they can represent the context in which the disease develops. Alternatively, they can be generated by a cause that is also at the basis of pemphigus, such as dysregulation of the immune system. In some cases, they could represent a trigger factor that induce epitope spreading phenomena leading to an autoimmune response.

### Thyroid diseases

Association between pemphigus and thyroid diseases has largely been reported, but published data are controversial. Some authors found that changes in the serum thyroid profile, positive titers of anti-thyroid autoAbs (anti-thyroid peroxidase and anti-thyroglobulin autoAbs) and Hashimoto’s thyroiditis were more frequent in PV patients than controls (175, 176) (Table 2). According to Parameswaran and coworkers, frequency of autoimmune thyroid disease was significantly higher in PV patients than in controls, and in PV patients’ relatives than in the control counterpart (177), in accordance with Heelan and coworkers that found a higher risk of hypothyroidism in PV subjects (178), mainly among women. However, the presence of laboratory thyroid alterations in PV patients not always correlates with clinical disease (176, 179). Recently, a large-scale study conducted on a cohort of 1985 PV patients found a significant association with Hashimoto’s thyroiditis in male patients but not in females, whereas no association was found between pemphigus and Grave’s disease and thyroid cancer (180).

### Malignancies

Several reports seem to indicate an association of non-paraneoplastic pemphigus with oncologic diseases, in particular with hematologic malignancies (181–184) (Table 2). According to Schulze and coworkers the percentage of pemphigus patients with hematologic malignancies was 3.9% (182). These findings were confirmed by Kridin and coworkers that found a higher prevalence of chronic leukemia (0.9% vs. 0.4%), multiple myeloma (0.8% vs. 0.4%) and non-Hodgkin lymphoma (1.8% vs. 1.2%) in pemphigus patients than in controls (183). While no definitive explanation has been suggested for this association, it has been proposed that the development of hematologic malignancies could be the result of chronic inflammatory stimulation or due to drug-induced immunosuppression. In this view, this disease could be a consequence rather than a trigger factor for pemphigus. In 1995, Ogawa and coworkers found a 5% prevalence of internal malignancies among PV patients, significantly higher than in the Japanese population (0.6%), according to their data, lung cancer was the most frequent form (185). More recently, Schulze and coworkers found a higher prevalence of oropharyngeal, gastrointestinal and colon cancer in PV patients than in controls (182). Large cohort studies confirmed the high prevalence of oesophageal and laryngeal cancers (respectively 3-fold and 2-fold higher in PV patients than in controls) but not of gastrointestinal and colon cancer (184) or lung cancer (186). As a pathogenetic hypothesis, solid malignant tumors could induce tissue alterations that lead to mucosal antigen unmasking and could favour the development of autoimmune response.

Kaposi’s sarcoma (KS) and pemphigus associations have been reported in the last decades as case reports or epidemiological studies (187–189). These studies lead to the hypothesis that HHV-8 could be a trigger for the onset of the autoimmune blistering disease. Furthermore, some authors described KS after the beginning of immunosuppressive therapy for pemphigus. Even if the link between the two diseases is still unclear, we may suppose that KS could be triggered by pemphigus immunosuppressive therapy and that pemphigus, such as other autoimmune blistering disease, could be triggered by the presence of HHV-8 in the skin cells.

### Autoimmune disorders

A high number of pemphigus patients has a familial history of autoimmune diseases. Several reports show a possible association of pemphigus with a second autoimmune disorder (5) (Table 2). This could be explained by the activity of the primary immune disease that could lead to the alteration of regulatory immune response inducing a second autoimmune condition. Moreover, autoimmune diseases could share the same signalling pathways, so the upregulation in one of these pathways could represent a predisposition to several autoimmune diseases.

A population-based large scale study by Kridin and coworkers stated that the prevalence of psoriasis was significantly greater in the patients with pemphigus than in the controls (respectively 3.3% vs. 1.2%) (190). Furthermore a population-based case–control study in Taiwan showed a greater prevalence rate of pemphigus in a population of 51,800 patients affected by psoriasis (191). The link between these two diseases is still unclear.

More reports link pemphigus to other autoimmune diseases such as lichen planus, systemic lupus erythematosus, pemphigoid, patchy alopecia areata and alopecia areata universalis, rheumatoid arthritis and autoimmune thyroiditis (5, 192–195).

## Conclusion

Although the understanding of pathogenic mechanisms of AIBDs has increased tremendously, there is still much to learn about factors affecting their onset, course, and therapy. Predisposing factors for pemphigus include genetic predisposition and comorbidities.

Precipitating factors, such as drugs, vaccines, pregnancy, radiations, emotional stress, infections, diet or other external factors, could induce pemphigus disease in the presence, but also in the absence of predisposing factors. However, in the majority of patients no conclusive trigger can be evaluated. In fact, most triggers reported in this review are not mechanistically confirmed. Thus, future studies should establish appropriate disease models and also investigate the relationship between trigger and predisposing factors with the aim to improve knowledge on pemphigus pathogenesis.

## Author contributions

FMo: Conceptualization, Writing – original draft. JS: Conceptualization, Writing – original draft. AS: Writing – original draft. LF: Writing – original draft. FMa: Writing – review & editing. AP: Writing – review & editing. BD: Writing – review & editing. GZ: Conceptualization, Supervision, Writing – original draft, Writing – review & editing.
